# Gene-Specific Signatures of Elevated Non-Synonymous Substitution Rates Correlate Poorly across the *Plasmodium* Genus

**DOI:** 10.1371/journal.pone.0002281

**Published:** 2008-05-28

**Authors:** Gareth D. Weedall, Spencer D. Polley, David J. Conway

**Affiliations:** 1 Department of Infectious and Tropical Diseases, London School of Hygiene and Tropical Medicine, London, United Kingdom; 2 Medical Research Council Laboratories, Fajara, Banjul, The Gambia; Pasteur Institute, France

## Abstract

**Background:**

Comparative genome analyses of parasites allow large scale investigation of selective pressures shaping their evolution. An acute limitation to such analysis of *Plasmodium falciparum* is that there is only very partial low-coverage genome sequence of the most closely related species, the chimpanzee parasite *P. reichenowi*. However, if orthologous genes have been under similar selective pressures throughout the *Plasmodium* genus then positive selection on the *P. falciparum* lineage might be predicted to some extent by analysis of other lineages.

**Principal Findings:**

Here, three independent pairs of closely related species in different sub-generic clades (*P. falciparum* and *P. reichenowi*; *P. vivax* and *P. knowlesi*; *P. yoelii* and *P. berghei*) were compared for a set of 43 candidate ligand genes considered likely to be under positive directional selection and a set of 102 control genes for which there was no selective hypothesis. The ratios of non-synonymous to synonymous substitutions (dN/dS) were significantly elevated in the candidate ligand genes compared to control genes in each of the three clades. However, the rank order correlation of dN/dS ratios for individual candidate genes was very low, less than the correlation for the control genes.

**Significance:**

The inability to predict positive selection on a gene in one lineage by identifying elevated dN/dS ratios in the orthologue within another lineage needs to be noted, as it reflects that adaptive mutations are generally rare events that lead to fixation in individual lineages. Thus it is essential to complete the genome sequences of particular species of phylogenetic importance, such as *P. reichenowi*.

## Introduction

Identifying genes under positive directional selection can help understand how parasites adapt to new survival or reproductive challenges. The dN/dS ratio (non-synonymous substitutions per non-synonymous site divided by synonymous substitutions per synonymous site) is commonly applied to scan for evidence of positive selection in comparative genomic analysis [1], [2]. Analyses of polymorphism among genome sequences of the human malaria parasite *P. falciparum* [3]–[5], and divergence between *P. falciparum* and the partially available genome sequence of the chimpanzee parasite *P. reichenowi* [3] show elevated dN/dS ratios in genes encoding membrane and exported proteins (considered to be under positive selection), as well as genes that are expressed at low abundance or at only one stage of the life cycle (considered to be under relaxed negative selection). However, the incompleteness of the *P. reichenowi* genome sequence (available sequence reads aligned to only ∼ 42% of the *P. falciparum* 3D7 genome sequence) means that most loci could not be effectively analysed for inter-specific divergence [3], so most signatures of positive directional selection have not yet been discriminated.

Pairwise analyses with other malaria parasite species may also identify loci under positive selection. However, given the great evolutionary distance between many of the species, such as between *P. falciparum* and the rodent parasite *P. yoelii* [6], studies of pairwise dN/dS suffer from too high a sequence divergence, causing synonymous substitutions to be saturated and making estimates of dN/dS rate ratios unreliable. Analyses of closely related species are preferable, and pairwise dN/dS analysis among the genomes of the rodent malaria parasites, *P. yoelii*, *P. berghei* and *P. chabaudi* [7], showed a similar overall trend to the *falciparum-reichenowi* analysis, with putative membrane proteins displaying higher dN/dS values than other genes. Could the results of that analysis (or analysis of other closely related species pairs such as *P. vivax* and *P. knowlesi*) be extrapolated to *P. falciparum* genes for which *P. reichenowi* orthologous sequences are not available? This study tests whether signatures from one clade of the *Plasmodium* genus can be used to predict those in other clades. The distributions of dN/dS values are compared for sets of orthologous loci in three phylogenetically independent species pairs, investigating a set of 43 candidate genes that are considered likely to be under positive selection and a set of 102 control genes for which there is no selective hypothesis.

## Results and Discussion

**Figure 1:**
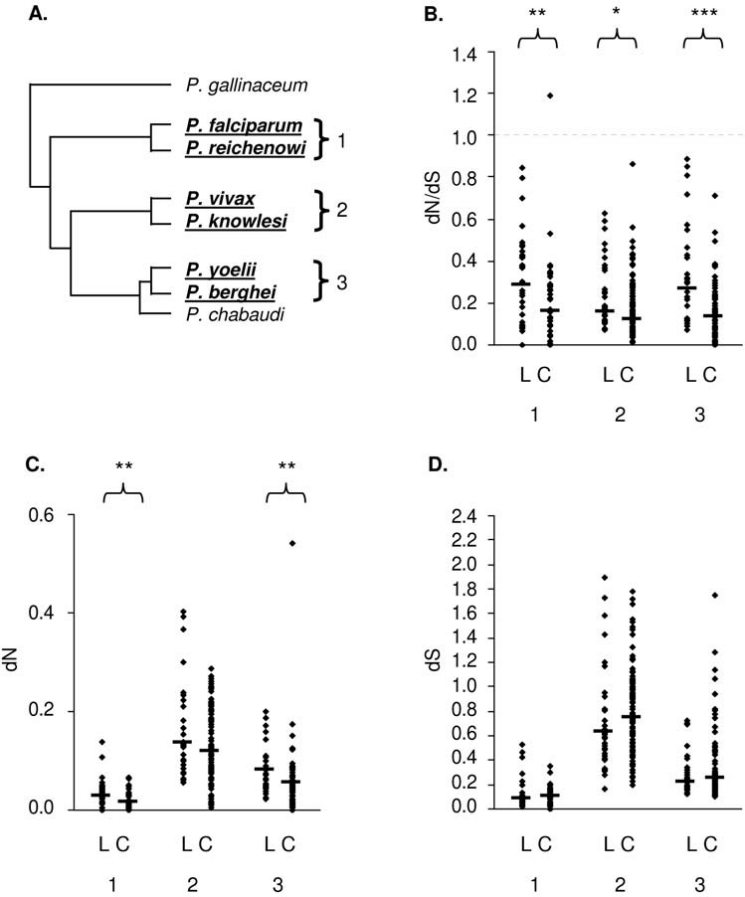
Genetic divergence among three pairs of *Plasmodium* species. A. Schematic representation of the phylogenetic relationship between sequenced *Plasmodium* genomes. Three pairs of closely related species (*falciparum-reichenowi*, *vivax-knowlesi* and *yoelii-berghei*) used for analysis are labelled clade 1, 2 and 3, respectively. (The phylogenetic position of *P. gallinaceum* in relation to the other species is not yet confirmed and awaits full genomic analysis, but is either an outgroup as illustrated here [10], [11] or more closely related to the *falciparum-reichenowi* clade). B. The distribution of dN/dS for candidate ligand genes and control genes (labelled ‘L’ and ‘C’) between species of each clade defined in panel A. Sample sizes were: clade 1, L = 33, C = 37; clade 2, L = 32, C = 92; clade 3, L = 29, C = 70. Asterisks indicate a significant difference between gene datasets by Wilcoxon's rank sum test (*<0.05, **<0.01, ***<0.001). C. The distribution of dN for the same loci. One extreme value (PY05686 vs. PB000528.03.0, dN = 8.06) is not shown. D. The distribution of dS for the same loci. Two extreme values (PY05686 vs. PB000528.03.0, dS = 45.69; PY02848 vs. PB100183.00.0, dS = 108.79) are not shown.

For each of the 43 candidate ligand genes analysed, inter-specific dN/dS ratios are shown for each of the three closely related species pairs, *P. falciparum / P. reichenowi*, *P. vivax / P. knowlesi*, and *P. yoelii / P. berghei* (Table 1, further details in table S1). To test whether this candidate ligand gene dataset is enriched in genes under positive selection, dN/dS values were compared with the control gene dataset (table S2) for each species pair (Fig. 1A) using Wilcoxon's rank sum test. For all three species pairs the median dN/dS ratio was significantly greater in the candidate ligand gene set than in the control set (*falciparum-reichenowi*, P = 0.0084; *vivax-knowlesi*, P = 0.0175; *yoelii-berghei*, P = 0.0003) (Fig. 1B). This was also seen for dN values (Fig. 1C), though not for dS (Fig. 1D), indicating a signature of positive selection on non-synonymous mutations leading to elevated dN/dS values in a proportion of the candidate ligand genes. Relaxed selective constraint could also result in elevated dN/dS, although there is no reason to expect that the candidate ligand genes should be under any less selective constraint than the control set of genes to maintain protein structure and function.

**Table 1:** A set of 43 candidate ligand gene loci with dN/dS ratios for three phylogenetically independent *Plasmodium* species pairs (*Pf/Pr*, *Pv/Pk* and *Py/Pb*)

*Pf* locus ID	Gene product	Evidence for ligand role	References	*Pf*/*Pr*	*Pv*/*Pk*	*Py*/*Pb*
PFB0310c	MSP4	D_a_, SE_m_	[20], [21]	0.84	0.48	-^n^
PFB0305c	MSP5	SE_m_	[21], [22]	0.80	0.59	-^n^
PF13_0201	TRAP	B, IIA, D_b_, AE_s_, SE_s_	[23]–[25]	0.70	0.35	0.72
MAL13P1.60	EBA140	B, IIA, AE_m_	[26]–[28]	0.57	0.42	-^n^
PF10_0352	MSP11	SE_m_	[29]	0.49	-^n^	-^n^
PF11_0486	MAEBL	B, IIA, D_b_, SE_s_, AE_m_	[30]–[32]	0.48	0.37	0.30
PFA0125c	EBA181	B, AE_m_	[33]	0.47	-^n^	-^n^
PFE0080c	RAP2	AE_m_	[34], [35]	0.44	-^n^	-^n^
PF10_0302	P28	IIA, D_b_, SE_o_	[36], [37]	0.43	-^n^	0.57
PF13_0248	P47	SE_g_	[38]	0.42	0.12	0.55
PFD1150c	RH4	AE_m_	[39]–[41]	0.42	-^n^	-^n^
PFD0210c	P36	D_b_	[42]	0.42	0.18	0.32
PF10_0303	P25	IIA, D_b_, SE_o_	[36], [37]	0.38	0.45	0.81
PF14_0102	RAP1	IIA, AE_m_	[34], [35], [43]	0.37	0.19	0.85
PFF0615c	Pf12	SE_m_	[44]	0.37	0.14	0.12
PFF0995c	MSP10	D_a_, AE_m_	[21], [45]	0.30	0.38	-^p^
PF11_0344	AMA1	B, IIA, D_a_, AE_m_/SE_m_, AE_s_/SE_s_	[46]–[48]	0.30	0.14	0.30
PFL0800c	celTOS	D_b_, AE_o_	[49]	0.26	0.63	-^n^
PF10_0344	GLURP	SE_m_	[50]	0.25	-^n^	-^n^
PFE0395c	Pf38	SE_m_	[44]	0.24	0.11	0.25
PFI1730w	CLAG9	D_c_, AE_m_	[51], [52]	0.24	-^n^	-^n^
PFC0640w	CTRP	D_b_, AE_o_	[53], [54]	0.24	0.25	0.34
PFC0210c	CSP	B, SE_s_	[55]–[57]	0.20	0.56	0.41
PFI1445w	RhopH2	AE_m_	[58]	0.18	0.07	0.10
PF13_0247	P48/45	D_d_, SE_g_	[59]	0.14	0.15	0.21
PFI0265c	RhopH3	D_a_, AE_m_	[60], [61]	0.11	-^n^	0.32
PFL2510w	CHT1	D_b_, AE_o(secreted)_	[62], [63]	0.10	0.14	0.13
PFE0075c	RAP3	AE_m_	[34]	0.09	-^n^	0.89
PFL0870w	PTRAMP	AE_m_	[64]	0.08	0.18	0.10
MAL7P1.208	RAMA	B, D_a_, AE_m_	[21], [65]	0.08	0.23	0.47
PFB0405w	P230	B, IIA, SE_g_	[66], [67]	0.08	0.11	0.23
PFC0120w	CLAG3.2	AE_m_	[68]	0.07	-^n^	-^n^
PFB0570w	SPATR	B, IIA, SE_s_	[69]	0.001	0.12	0.09
PF08_0003	TryThrA	IES	[70]	-^n^	-^p^	0.10
PF14_0040	SOAP	D_b_, AE_o_	[71]	-^n^	0.56	-^n^
PF13_0338	Pf92	D_a_, SE_m_	[21], [44]	-^n^	0.27	-^n^
PF08_0136b	WARP	AE_o_	[72]	-^n^	0.26	0.43
PFD0215c	P36p	D_b_, SE_s_	[42]	-^n^	0.25	0.30
PFE0120c	MSP8	SE_m(in *P. yoelii*)_	[73]	-^n^	0.18	0.12
PFI1145w	PLP3/MAOP	D_b_, AE_o_	[74]	-^n^	0.17	0.13
PFL1385c	MSP9	B, SE_m_	[75], [76]	-^n^	0.17	0.07
PFD0240c	Pf41	SE_m_	[44]	-^n^	0.16	0.30
PFC0420w	CDPK3	D_b_, AE_o_	[77], [78]	-^n^	0.08	0.18

B = binding assay. IIA = invasion inhibition assay. D = gene disruption experiment which either (_a_) could not produce viable parasites in asexual culture; (_b_) reduced or abolished the traversal of cell membranes or tissue layers; (_c_) abolished receptor binding; or (_d_) reduced fertilization. SE/AE/IES = surface/apical/infected erythrocyte surface expression at (_s_) sporozoite, (_m_) merozoite, (_g_) gametocyte, or (_o_) ookinete stage. -^n^ unambiguous orthologues could not be identified in one or both species; -^p^ orthology could not be resolved among alternative possible orthologues. For each gene a maximum of three references are given. Twelve other candidate ligand loci could not be analysed due to complex sequence evolution (see Methods).

It should be noted that analysis of any single one of these genes in isolation would not lead to a strong conclusion of positive selection, since none showed a dN/dS value >1. Inter-specific dN/dS values for whole genes are hardly ever >1 even when positive selection occurs, due to the effect of negative background selection on many sites within most genes [1], [2], so comparison of relative dN/dS values across sets of genes is a more sensitive way of scanning for evidence of positive selection than searching for individual values above 1 or any other arbitrary cut off.

**Figure 2:**
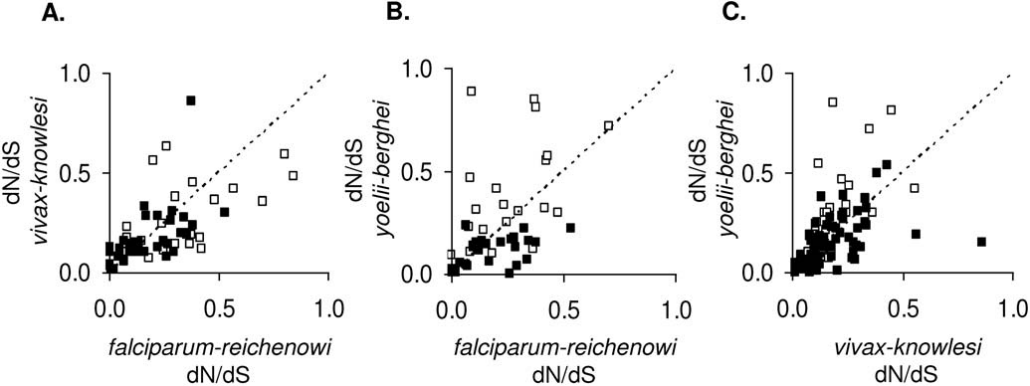
Scatterplots of dN/dS estimates for orthologous loci in independent *Plasmodium* species pairs. A. *vivax-knowlesi vs. falciparum-reichenowi*, B. *yoelii-berghei vs. falciparum-reichenowi* and C. *yoelii-berghei vs. vivax-knowlesi*. A line of identity representing equal selective constraint and/or positive selection in orthologous genes in different species is shown on each plot (dotted line). Filled squares represent gene pairs from the control gene dataset, open squares gene pairs from the set of candidate ligand genes. Sample sizes and results of Spearman's rank correlation analysis are shown in Table 2.

To assess the predictive power of dN/dS across the *Plasmodium* genus, rank correlations (Spearman's *ρ* = rSp) were applied to test whether similar relative selective forces operate on orthologous genes in different species. Table 2 shows the correlation of dN/dS, dN and dS indices for all genes among the three different species pairs. Pairwise scatterplots of dN/dS values are shown in Fig. 2. The predictive power is quantified by r^2^Sp which represents the amount of variability in one axis which can be explained by variability in the other. dN/dS was significantly, though poorly, positively correlated between independent species pairs for both candidate ligand genes and control genes. Correlations were greater for dN than for dS, supporting the idea that selection affects the correlations while synonymous substitutions are mostly stochastic. However, the predictive power of dN/dS for one species pair on another is lower for candidate ligand genes (25 %, 21 % and 31 % for *Pf/Pr* versus *Pv/Pk*, *Pf/Pr* versus *Py/Pb*, and *Pv/Pk* versus *Py/Pb* respectively) than for control genes (55 %, 35 % and 44 % for the respective three comparisons). This indicates that the correlation is not improved by positive selection but is actually made worse. Discrete processes of positive selection will have occurred in different species lineages, against a background of selective constraint that varies among genes in a manner that is apparently more homogeneous between different lineages.

**Table 2:** Spearman's rank correlation (rSp) of pairwise sequence divergence estimates for orthologous loci among different species pairs

Species pairs compared	Gene dataset	N	Index	rSp	r^2^Sp	
*falciparum-reichenowi* versus *vivax-knowlesi*	Ligand	23	dN/dS	0.50	0.25	*
			dN	0.56	0.32	**
			dS	0.04	0.002	
	Control	35	dN/dS	0.74	0.55	***
			dN	0.76	0.58	***
			dS	0.49	0.24	**
*falciparum-reichenowi* versus *yoelii-berghei*	Ligand	21	dN/dS	0.46	0.21	*
			dN	0.41	0.17	
			dS	−0.01	0.0002	
	Control	26	dN/dS	0.59	0.35	**
			dN	0.39	0.15	*
			dS	−0.19	0.03	
*vivax-knowlesi* versus *yoelii-berghei*	Ligand	25	dN/dS	0.56	0.31	**
			dN	0.54	0.30	**
			dS	0.25	0.06	
	Control	67	dN/dS	0.66	0.44	***
			dN	0.54	0.29	***
			dS	0.38	0.15	**

N = number of gene loci analysed for the pairwise correlations between each independent species pair. ^*^ P<0.05, ^**^ P<0.01, ^***^ P<0.001

Thus, although broadly similar signatures indicating positive selection on distinct classes of genes may be seen in different parts of the *Plasmodium* phylogeny, predictions about positive selection on individual genes for which sequence data are currently missing in particular species cannot be reliably extrapolated from orthologues in other parts of the phylogeny. To detect loci that have undergone positive directional selection in the lineage of a particular species, sequences must be directly compared with orthologues of a closely related species. As *P. falciparum* is currently the most important human parasite, completion of the closely related *P. reichenowi* genome sequence should now have particularly high priority [3].

## Materials and Methods

### Sets of candidate genes and controls

A set of 55 single-locus genes encoding surface proteins that are putatively ligands at various life cycle stages was first defined. These genes are candidates to display signatures of positive selection due to their likely role in host-parasite interaction, and of these, 43 could be included in comparative dN/dS analyses as noted in the following section. Loci in this candidate gene dataset were compared with loci from a control dataset chosen to represent an unbiased sample of genes not hypothesised to be under positive selection. The control set was of loci on *P. falciparum* chromosome 3 that contained one or more nucleotide difference among the sequences of five isolates as published [8] with data searchable on PlasmoDB (www.plasmodb.org) [9]. Of the 104 such loci identified, two (PFC0210c and PFC0420w) were already included in the candidate ligand gene dataset and were thus excluded from the control dataset, which therefore consisted of 102 genes.

### Defining orthologous genes for analysis of sequence divergence between species

Pairwise nucleotide divergence was estimated for 3 pairs of closely related species: *P. falciparum* and *P. reichenowi*; *P. vivax* and *P. knowlesi*; and *P. yoelii* and *P. berghei* (Fig. 1A). Two other species for which genome sequence data are available, *P. gallinaceum* and *P. chabaudi*, were not included in the present analysis as the former is not very closely related to any other species [10]–[13], and the latter would add little extra information to the *yoelii-berghei* pair [7]. Protein-coding gene sequences in the *P. falciparum* 3D7 genome sequence (release date 11/02/2005), produced by a consortium of the Wellcome Trust Sanger Institute (WTSI), the Institute for Genomic Research (TIGR) and Stanford University [14], were downloaded from the PlasmoDB website (http://www.plasmodb.org/common/downloads/) [9]; sequences from *P. vivax* (release date 03/11/2005) and *P. yoelii* (23/07/2004) [15], produced by the Institute for Genomic Research (TIGR), were downloaded from the TIGR website (ftp://ftp.tigr.org/pub/data/Eukaryotic_Projects/); shotgun sequences from *P. reichenowi* (11/03/2004) [3], and gene sequences from *P. knowlesi* (06/01/2006) and *P. berghei* (08/06/2004) [7] were produced by the Wellcome Trust Sanger Institute and were downloaded from the WTSI website (ftp://ftp.sanger.ac.uk/pub/pathogens/).

Orthologues to *P. falciparum* predicted protein sequences were defined by BLASTp (protein vs. protein) searches against databases of *P. yoelii*, *P. berghei*, *P. vivax* and *P. knowlesi* predicted proteins, and required a reciprocal best match against the *P. falciparum* predicted protein database. For added stringency, each pair of putative orthologues (*yoelii-berghei*, *vivax-knowlesi*, *falciparum-reichenowi*) were BLASTed against the database of the other species of the pair to ensure that the best matches to the *P. falciparum* sequences in each species were also reciprocal best matches to each other. Where this was not the case the pair was not analysed (detailed results of BLAST searches are shown in tables S1 and S2).

No database of predicted proteins existed for *P. reichenowi*, so *P. falciparum* predicted protein sequences were used to search the *P. reichenowi* genomic contig database using tBLASTn (protein versus DNA translated in all 6 possible reading frames). In a number of cases where *P. reichenowi* orthologues could not be identified in the contig data, published *P. reichenowi* sequences were obtained from GenBank or sequences built from shotgun sequencing reads were used (table S3). For each gene, the *P. falciparum* coding sequence (introns excluded) was aligned to the best matching *P. reichenowi* contig using the SeqMan II program (DNASTAR, Madison, WI) to define the start and end of the coding sequence and the intron-exon boundaries. *P. reichenowi* contig sequences contained some regions of single-read coverage, so nucleotide mismatches in regions of single-read coverage were edited to match the *P. falciparum* sequence, and only well supported nucleotide mismatches in regions of multiple-read coverage were used for analysis. If a *P. reichenowi* gene sequence contained apparent frameshifts supported by multiple-read coverage, it was considered to be a pseudogene and not analysed.

Forty three of the 55 candidate ligand gene loci examined could be analysed for pairwise divergence between orthologues. Twelve loci (*msp1*, *msp2*, *msp3*, *msp6*, *msp7*, *eba175*, *eba165*, *ebl1*, *rh1*, *rh2a*, *rh2b*, *rh3*) were not analysed, because (i) unambiguous orthologues could not be defined, or (ii) molecular evolution appeared complex such that dN and dS may not represent the accumulation of substitutions between species (some genes had dimorphic alleles that were more divergent than the paired species sequence, and others showed evidence of gene conversion with paralogues), or (iii) an orthologue appeared to be a pseudogene. Of the 102 control gene loci, all orthologous pairs identified were analysed unless they contained a pseudogene. The relatively low number of *falciparum-reichenowi* gene pairs analysed (37 of 102 control genes, compared to 92 and 70 for the other species pairs) reflects the low sequence coverage of the *P. reichenowi* genome to date.

### Analysis of pairwise between-species dN and dS values for individual genes

Orthologous protein sequence pairs were aligned using clustalW [16] and the protein alignments imposed upon the nucleotide sequences using the program pal2nal [17]. For each sequence pair, pairwise dN, dS and dN/dS indices were estimated by maximum likelihood using the codeml program [18]. Maximum likelihood estimates of dN/dS were used since they are more accurate than approximate methods such as the Nei-Gojobori method when transition/transversion rate biases and nucleotide composition or codon frequency biases exist [19]. Three independent runs of codeml were made with different initial estimates for the transition/transversion rate ratio (*k*) and the dN/dS ratio (run 1: *k* = 1, dN/dS = 1; run 2: *k* = 0.1, dN/dS = 10; run 3: *k* = 10, dN/dS = 0.1) so that each run began at a different point in the likelihood space. If different final results were obtained between runs, those with the highest log likelihood value (lnL) were used, lower lnL values being assumed to represent local likelihood optima. Non-parametric statistical tests, Wilcoxon's rank sum test and Spearman's rank correlation, were carried out using STATA 9 (StatCorp LP, Texas, USA) as the indices of divergence were not assumed to be normally distributed.

## Supporting Information

Table S1 Results of BLAST sequence similarity searches to determine orthologuous pairs of loci in six Plasmodium genomes for 43 candidate ligand genes
(0.08 MB XLS)

Table S2 Results of BLAST sequence similarity searches to determine orthologuous pairs of loci in six Plasmodium genomes for 102 genes on P. falciparum chromosome 3.
(0.12 MB XLS)

Table S3 Shotgun sequencing reads used to build 7 of the P. reichenowi gene sequences
(0.03 MB DOC)
