# Supplementary material for: Gene-Specific Signatures of Elevated Non-Synonymous Substitution Rates Correlate Poorly across the Plasmodium Genus
Source: PLoS One. 2008 May 28;3(5):e2281. doi: 10.1371/journal.pone.0002281 (PMC2384006; doi:10.1371/journal.pone.0002281)
Supplement: Table S3 — Shotgun sequencing reads used to build 7 of the P. reichenowi gene sequences (0.03 MB DOC) [file pone.0002281.s003.doc]

**Table S3.** Shotgun sequencing reads used to build 7 of the *P. reichenowi* gene sequences

| Gene product | Shotgun sequence reads |
| --- | --- |
| MSP4 | reich886a05.p1k, reich1009b04.q1k, reich204g05.q1k, reich132h11.q1k  reich204g05.p1k |
| MSP5 | reich345e05.q1k, reich432f10.q1k, reich345e05.p1k, reich1182a03.p1k |
| MSP11 | reich298h02.q1k, reich249f07.q1k, reich393a07.q1k, reich158a05.p1k  reich249f07.p1k |
| RAP2 ^a^ | reich91f07.q1k, reich581b11.q1k, reich301f10.q1k, reich534h06.p1k |
| RAP3 | reich308g09.p1k, reich451f07.p1k, reich29g09.q1k, reich1212e05.q1k  reich451g08.q1k, reich308g09.q1k |
| PTRAMP | reich471f10.q1k, reich221a04.q1k, reich320b09.q1k, reich331e06.p1k  reich471f10.p1k |
| CTRP | reich067c05.p1k, reich067c05.q1k, reich154c02.p1k, reich154c02.q1k  reich380c02.p1k, reich380c02.q1k, reich415g05.p1k, reich415g05.q1k  reich437d09.p1k, reich437d09.q1k, reich704e04.q1k, reich723e12.p1k  reich819g10.q1k, reich819g10.p1k, reich862b09.p1k |

^a^ a portion of sequence was also generated by PCR from *P. reichenowi* genomic DNA to complete the coding sequence
